# Acute Esophageal Necrosis Associated With Methicillin-Resistant Staphylococcus Aureus Septicemia: A Case Report

**DOI:** 10.7759/cureus.8720

**Published:** 2020-06-20

**Authors:** Yasser M Bawazir, Mohammad A Mustafa

**Affiliations:** 1 Internal Medicine: Rheumatology, King Abdulaziz University, Jeddah, SAU; 2 Internal Medicine: Rheumatology, University of Jeddah, Jeddah, SAU

**Keywords:** acute esophageal necrosis, staphylococcus aureus, gastrointestinal bleeding

## Abstract

Acute esophageal necrosis (AEN) is a multifactorial disease with a predilection for elderly male patients with multiple medical problems, including mainly hypoperfusion and malnutrition. The diagnosis is confirmed by biopsy, and AEN is managed conservatively by controlling the underlying illness. We report a case of a 65-year-old man with malnutrition, heart failure secondary to ischemic heart disease, infection with methicillin-resistant Staphylococcus aureus septicemia, and positive herpes simplex serology, who developed upper gastrointestinal (GI) bleeding. Endoscopy confirmed AEN, and he was managed conservatively with antacids, hydration, and antibiotics.

## Introduction

Acute esophageal necrosis (AEN), also known as Gurvits syndrome, was first described by Goldenberg in 1990 and was renamed distinctive pathology by Gurvits in 2007 [1-2]. The incidence of AEN ranges from 0.01% to 2% with male predominance, and the mean age of presentation is 68 years [3-4]. The reported mortalities in 30% of cases were most likely related to underlying medical illnesses [3,5]. The universal presentation of this group of patients is upper gastrointestinal (GI) bleeding in the form of coffee-ground vomitus or melena, which can be associated with abdominal pain, nausea, vomiting, dysphagia, fever, and syncope. AEN can arise in a patient with hypoperfusion, hyperglycemia, malnutrition, and infections with viral and bacterial pathogens [3].

## Case presentation

This is a case of a 65-year-old Saudi man with known type 2 diabetes mellitus, hypertension, ischemic heart disease, heart failure, with an ejection fraction of 45%, and a 30-pack/year smoking habit, which he quit 10 years ago. He had a history of a large perianal abscess complicated by a vesicocutaneous fistula (VCF) due to the backpressure of the abscess, which required surgical resection with colostomy insertion 4 months ago. Subsequently, he became bedridden. He was brought to the emergency department, presenting with a history of altered mental status, fatigue, decreased oral intake, and fever for one week. His medications included atorvastatin 40 mg daily, aspirin 81 mg daily, furosemide 40 mg daily, bisoprolol 2.5 mg daily, insulin lispro pre-meal three times daily, and basal insulin detemir once daily. The patient denied alcohol and drug intake.

He appeared malnourished and drowsy upon presentation to the emergency department. His Glasgow coma scale (GCS) was 14, and his vital signs were as follows: temperature at 38°C, blood pressure at 113/60, respiratory rate at 18, and heart rate at 120. The oxygen saturation of room air was 96%. The following were noted upon examination: normal heart sounds, no murmurs, jugular venous pressure (JVP) not raised, bilateral lower limb pedal pitting edema, bilateral equal breathing sound with minimal fine inspiratory basal crackles, no tenderness or guarding in the abdomen, clean colostomy stoma, no active arthritis or skin rashes, and Stage III bedsore on the sacral area with granulation tissue and yellowish discharge.

Initial hematological examination revealed white blood cells count (WBC) 21,000 cells/µL with left shift, hemoglobin 12 g/dL, platelets 236,000 u/L, normal coagulation profile, glucose 9.1 mmol, creatinine 130 µmmol/L, blood urea nitrogen (BUN) 20 mmol/L, Na 131 mmol/L, K 3.4 mmol/L, Cl 91 mmol/L, lactic acid 6.5 mmol, albumin 15 g/L, normal liver function tests, normal cardiac enzymes and electrocardiogram, C-reactive protein (CRP) 303 mg/L, and urine analysis showed positive white blood cell (WBC), trace proteins, and positive nitrate. Additionally, his COVID-19 polymerase chain reaction test was negative, and brain computed tomography (CT) showed no acute brain insults. Blood, urine, and wound cultures were also taken. The toxicology screen was negative. The patient was stabilized, received proper hydration, and broad-spectrum antibiotics were initiated. Subsequently, his urine and blood cultures came positive for methicillin-resistant (MRSA) Staphylococcus aureus (MRSA S. aureus) 48 h later, and vancomycin was initiated.

Searching for the source of the bacteria, an echocardiogram was done to rule out infective endocarditis. It showed an ejection fraction of 30%, mild dilated left atrium, mild mitral valve regurgitation, and no vegetation. Transesophageal echocardiogram (TEE) was negative for vegetation. Magnetic resonance imaging of the lumbosacral area showed subcutaneous edema with enhancement and no collections or features of osteomyelitis.

**Figure 1:**
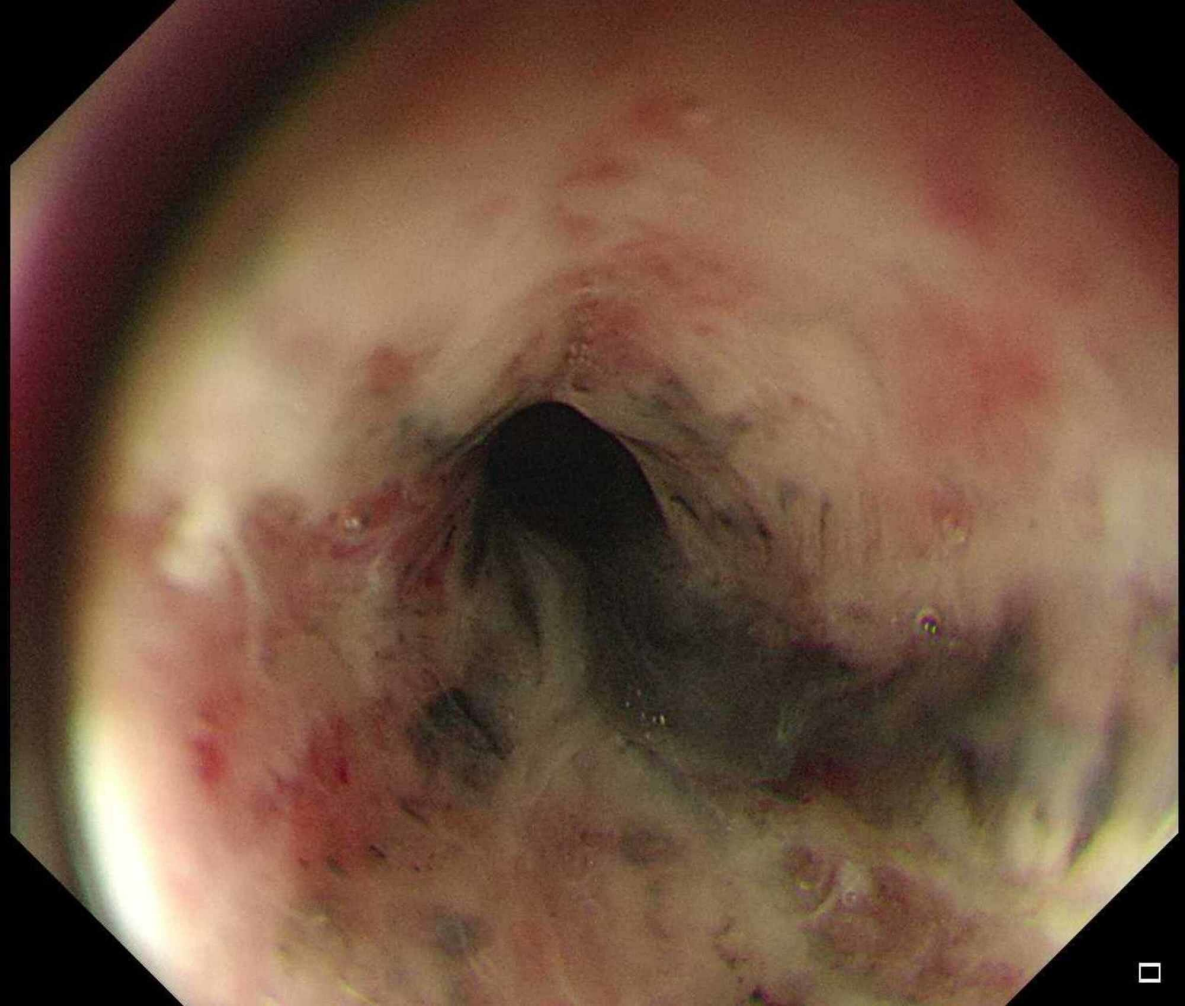
Esophagogastroduodenoscopy revealed black necrotic tissue in the distal part of the esophagus.

**Figure 2:**
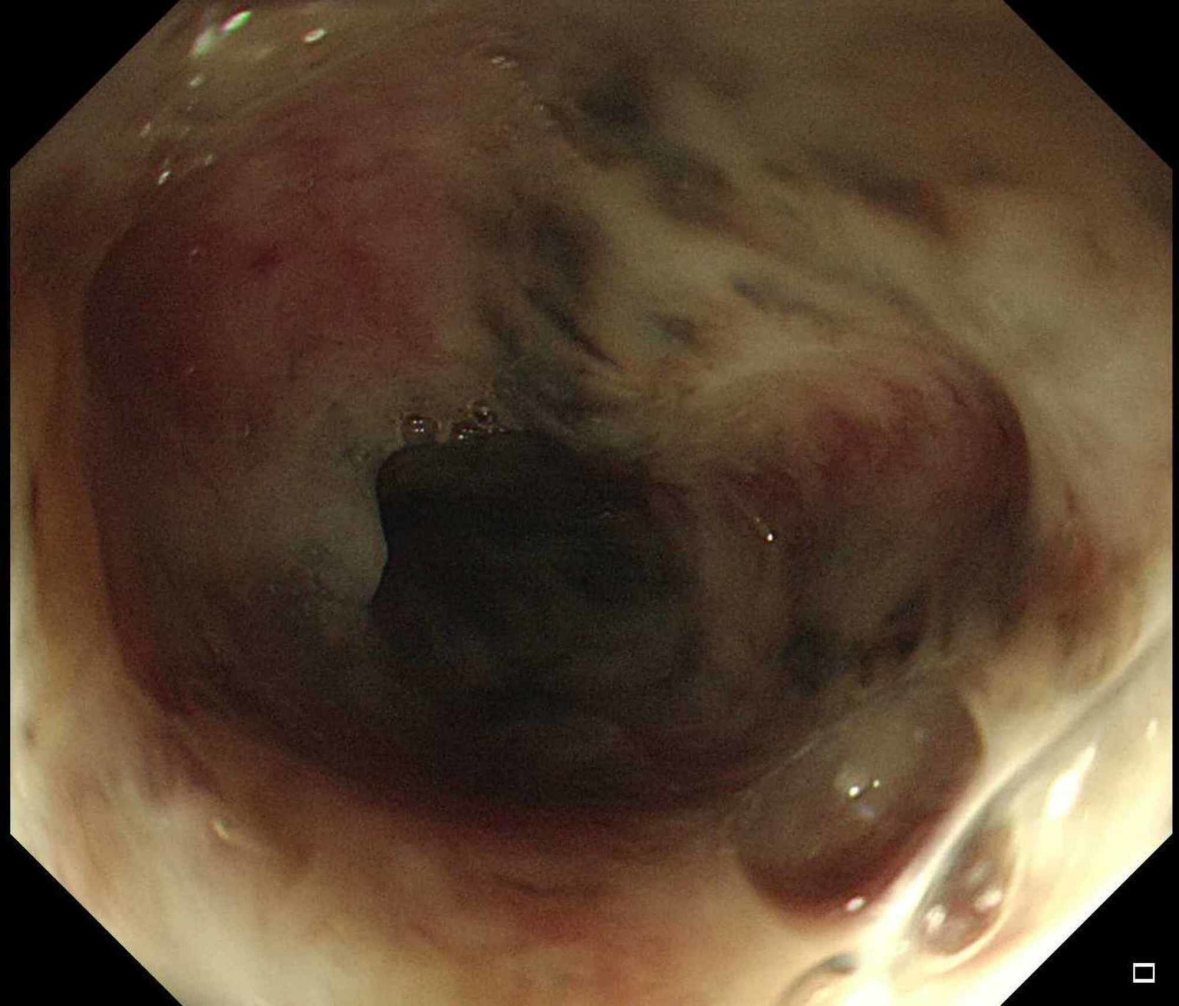
Esophagogastroduodenoscopy revealed black necrotic tissue in the distal part of the esophagus.

**Figure 3:**
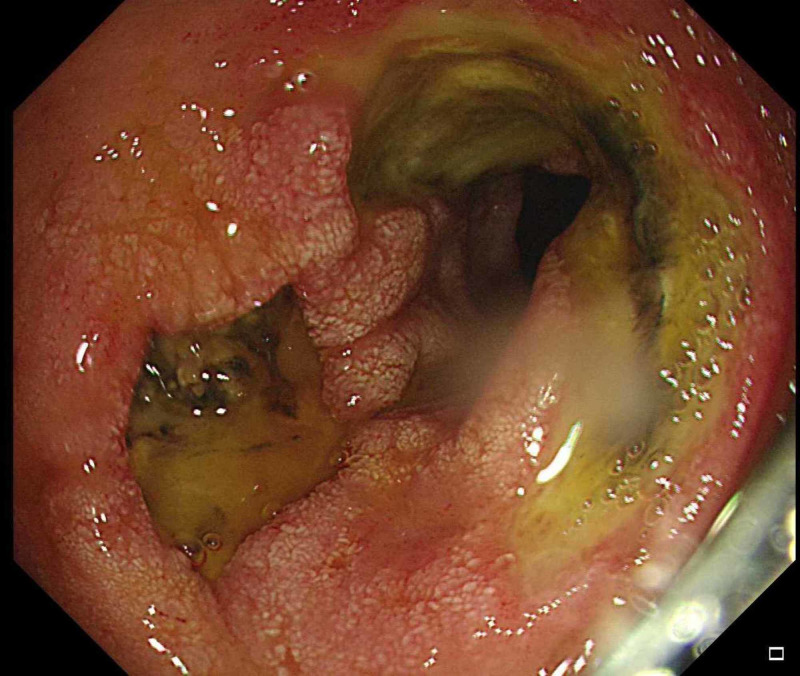
Two duodenal ulcers

On the fourth day of admission, he developed melena through the colostomy. His hemoglobin dropped to 7 g /dL, his coagulation profile was normal, and there were no significant changes from the previous workup. Endoscopy showed black pigmentation at the distal end of the esophagus with two duodenal ulcers (Figures 1-3). The biopsy was not taken because of extensive necrosis and possible perforation. The gastroenterology team suggested a workup for vasculitis, ischemia, cytomegalovirus (CMV), and herpes simplex (HSV). Proton pump inhibitor (PPI) infusion was initiated.

Rheumatological assessment for vasculitis and other autoimmune disorders was unremarkable. There were no constitutional symptoms, although there was bilateral lower limb edema. He was negative for antinuclear antibody (ANA), rheumatoid factor (RF), anti-citrullinated peptide antibody (Anti CCP), cytoplasmic antineutrophil cytoplasmic antibody (C-ANCA), perinuclear anti-neutrophil cytoplasmic antibody (P-ANCA), cryoglobulins, complements, and antiphospholipid antibodies. A repeated blood culture came positive for methicillin-resistant Staphylococcus aureus (MRSA), CRP was 103 mg/L, and lactate was 2.6 mmol. Viral serologies for human immunodeficiency virus, hepatitis B and C viruses, and cytomegalovirus (CMV) were negative. Herpes simplex virus (HSV) immunoglobulin M (IgM) antibody came positive. An infectious disease team was consulted, and they suggested starting acyclovir to treat his HSV esophagitis.

CT angiography was performed to confirm vasculitis. There was no evidence of bowel ischemia, and there were moderate atherosclerotic changes of the aorta and its branches. There was a filling defect in the right common iliac and in the right internal iliac arteries consistent with thrombosis. There was no evidence suggestive of vasculitis. Hematology was consulted regarding anticoagulation, and therapeutic heparin was initiated, as his thrombus was provoked by immobilization. Additionally, monitoring for bleeding signs was advised because of the high risk of bleeding from the duodenal ulcers. Malignancy surveillance was negative.

While on PPI, antibiotic, and antiviral treatments, the patient improved, and his melena stopped on the third day after endoscopy. Antibiotic use was continued for 14 days after the first negative blood culture, as well as acyclovir, for a total of 14 days as suggested by the infectious disease team. The patient was later discharged, and he retained full consciousness.

## Discussion

We reported this case of acute esophageal necrosis, wherein MRSA septicemia was the underlying etiology. Several organisms have been reported to be responsible for acute esophageal necrosis such as Klebsiella pneumonia, Penicillium chrysogenum, HSV, CMV, candida, and other fungal organisms. However, to the best of our knowledge, there has been no previous report on MRSA septicemia causing AEN [3,6]. Our patient has multiple risk factors such as atherosclerosis, heart failure, poorly controlled diabetes, hypoalbuminemia, hypertension, and sepsis, all of which can affect the tissue perfusion of the distal end of the esophagus and can precipitate acute esophageal necrosis, which are consistent with literature description of acute esophageal necrosis, also known as Gurvits syndrome [3]. Vasculitis specifically polyarteritis nodosa and antiphospholipid were suspected, and the workup was negative in our case.

Several hypotheses discussed the pathogenesis of this disease. Gurvits et al. suggested what was known as the “two-hit hypotheses.” The upper two-thirds of the esophagus has an abundant blood supply, while the lower third of the esophagus has a low prolific blood supply, making it more liable for hypoperfusion in a patient with poor hemodynamics and hence a higher risk for necrosis. A repeated mucosal injury from gastric acid reflux and impaired mucosal defensive mechanisms will increase the tissue damage [3,7].

The risk factors for AEN were hypoperfusion and shock being the most common, malnutrition, sepsis, diabetic ketoacidosis, renal impairment, alcohol intake, gastric volvulus, traumatic transection of the aorta, polyarteritis nodosa, and hematologic malignancy [3-4]. Thromboembolic phenomena and coagulopathy associated with malignancy can precipitate tissue injury, leading to AEN. The universal presentation of this group of patients is upper GI bleeding, which can be associated with abdominal pain, nausea, vomiting, dysphagia, fever, and syncope [3,8]. In a multicenter case series of eight cases, hyperglycemia was determined in 80% of the cases [9].

Endoscopy will show a circumferential black mucosa extending from the gastroesophageal junction with variable length. Performing a biopsy is controversial, as it may cause perforation and is not required for confirming the diagnosis, although it reveals the necrotic epithelial cell [10]. There are several reports on stenosis, stricture, perforation, and mediastinitis occurring as complications of this disease [3].

The management of this illness involves treating the underlying illness, proper hydration, and the use of proton pump inhibitors. Sucralfate was used in some cases because of its mucosal protective action. Surgical intervention was required for complicated cases involving obstruction, stricture, and perforation [3-4,9].

The prognosis of AEN is poor with a high mortality rate, which is mostly related to underlying illnesses or related to complications such as stricture or perforation. However, the mortality of AEN alone is only 6% [3,8].

## Conclusions

Acute esophageal necrosis is a rare disease, which is predominant in men with a median age of 68 years with the following risk factors: hypoperfusion, hyperglycemia, malnutrition, renal impairments, malignancy, gastric obstruction, and sepsis. The diagnosis is confirmed by an endoscopic evaluation that shows black necrotic tissue in the lower third of the esophagus. Biopsy is not required to confirm the diagnosis, and it carries a risk of perforation. AEN is managed conservatively with antacids, hydration, and treatment of the underlying illness.
